# Surgical ligation of filum terminale type IV arteriovenous fistula with giant venous varix

**DOI:** 10.3171/2025.7.FOCVID2536

**Published:** 2025-10-01

**Authors:** George W. Koutsouras, Raahim Bashir, Grahame Gould

**Affiliations:** Department of Neurosurgery, SUNY Upstate University Hospital, Syracuse, New York

**Keywords:** spinal arteriovenous fistula, indocyanine green, clipping, microsurgery, filum terminale

## Abstract

The authors present the case of a type IVc filum terminale arteriovenous fistula in a female patient in her 40s, who presented with lower abdominal pain, back pain, and lower extremity weakness. MRI showed an intradural mass with serpiginous vessels. Spinal angiography revealed a type IVc perimedullary fistula primarily fed by the anterior spinal artery with a giant venous varix. Because of anatomical factors, endovascular embolization was not feasible, and surgical ligation was performed via laminectomy at L1–3. The fistula seen within the filum terminale was successfully clipped, with no further venous outflow on angiography. The patient recovered with complete symptom improvement.

The video can be found here: https://stream.cadmore.media/r10.3171/2025.7.FOCVID2536

## Transcript

We describe a surgical ligation of a filum terminale type IV arteriovenous fistula with a giant venous varix.

### 0:27 Patient Presentation.

A previously healthy female in her 40s presented to the emergency department with acute back pain radiating to her lower abdomen after 3 weeks of intermittent episodes of bilateral extremity numbness and gait difficulty.

Her neurological examination demonstrated 4/5 right quadriceps weakness, bilateral lower extremity numbness, and a wide-based gait. Initial workup in the ED included a CT abdomen with IV and PO contrast revealing a 2-cm hyperattenuating lesion in the spinal canal at the L2 level.

### 0:56 Neurological Imaging.

Subsequent workup included lumbar spine MRI which demonstrated an extramedullary intradural mass with prominent, associated serpiginous vessels spanning from T12 to L3, followed by spinal catheter angiogram.

### 1:10 Spinal Angiogram.

Spinal angiography demonstrated an arteriovenous fistula filling from the left L2 lumbar segmental artery to the artery of Adamkiewicz and early venous drainage into the venous varix. Multiple small feeding arteries can be visualized, and venous drainage extends cranially and caudally with marked ectasia extending into the epidural venous plexus. These findings are consistent with a Takai type IVc perimedullary arteriovenous fistula at L2.

### 1:37 Spinal AVF Types.

Various classification schema for spinal arteriovenous fistulas have been described. The Takai schema, published in 2017, is appealing for its simplicity and consistency with prior classification schema.^1,2^ It divides spinal arteriovenous fistulas into 5 types based on location, size, venous outflow, and feeding arteries. As previously noted, this patient’s fistula is consistent with a type IVc Takai lesion based on its perimedullary location, presence of multiple feeders, and giant fistula.

### 2:06 Rationale for the Procedure.

Given the patient’s young age, good health, and culprit neurological signs and symptoms, treatment of the fistula was recommended. Thus, options for treatment were discussed including surgical ligation versus endovascular embolization. Radiosurgery was not offered. The long and tortuous course of the feeding arteries and small size of those vessels were deemed a high risk for endovascular navigation with perceived low likelihood of achieving adequate microcatheter position near the fistulous point necessary for safe and curative embolization. Therefore, open surgical ligation was considered a safer, definitive option for this patient

### 2:39 Risks of the Procedure and Potential Benefits.

Risks and benefits of surgery, as discussed with the patient, included bleeding, infection, nerve root or conus injury with associated neurological deficit, and CSF leak. Benefits included resolution of congestion myelopathy with improvement in neurological symptoms and durable treatment of the fistula.

### 2:59 Setup: Equipment.

In preparation for the procedure, the following instruments were made available. Intraoperative ultrasound with a cranial guidance transducer for confirmation of the venous varix prior to the opening the dura, standard laminectomy and microdissectors, IV indocyanine green for anesthesiology infusion for video angiography, microvascular Doppler, aneurysm clips, and intraoperative neuromonitoring.

### 3:20 Setup: Positioning for L1–3 Laminectomy With Dural Opening.

After informed consent was obtained, the patient was brought to the operating room where she was positioned in the prone position on an open Jackson frame. A spinal needle was used to localize the planned L1–3 laminectomy under fluoroscopy, and the corresponding midline incision was marked.

### 3:34 Key Surgical Steps Explained.

Upon completion of the laminectomies and dural opening, arachnoid dissection was completed to isolate the pathologic arterial and venous structures from the surrounding neurological tissue. Temporary clip ligation was performed prior to cauterizing and dividing vessels to ensure no changes in neuromonitoring. This was followed by visualization of the filum terminale with permanent clip ligation of the venous outflow.

### 3:58

Following completion of the L1–3 laminectomies, ultrasound confirmed appropriate exposure to the cranial and caudal poles of the venous varix. The dura was opened in a linear fashion and tacked up using 4-0 Nurolon suture, in standard fashion.

### 4:08 Microsurgical Dissection of Fistula.

Utilizing our angiographic understanding of the fistula, the small feeding arteries and arterialized venous complex were dissected using microinstruments. The majority of the arterial feeders were found on the patient’s right side, despite the expectations related to the angiogram. Following the dissection of the key arterial and venous structures, IV ICG videoangiography was performed, identifying early fluorescence of a suspected arterial feeder and subsequent fluorescence in the arterialized venous varix and ectatic veins.

### 4:39

Coinciding with the ICG videoangiogram you can visualize the arterial feeder into the venous varix with immediate filling of the subsequent arterialized venous outflow. Following ligation of that identified superficial feeding artery, persistent AV shunting remained on micro-Doppler. Further dissection around the nerve rootlets identified 4 additional arterial feeders on the cranial side of the varix. These were subsequently clip ligated and disconnected following no neuromonitoring changes seen. Despite this, micro-Doppler and ICG angiography demonstrated persistent arterialized flow in the varix and deeper dissection was pursued.

### 5:11 Filum Terminale Identification and Permanent Ligation of Fistula.

Finally, after further dissection of the varix was performed cranially and caudally, a hypervascular structure, subsequently confirmed as the filum terminale, was noted to be attached to the deep aspect of the venous varix cranially, with AV shunting evident directly within the filum. The filum was directly stimulated without any neuromonitoring response. The filum was then identified on the caudal side, and a permanent clip was placed incorporating the caudal venous outflow for occlusion of the fistula and division of the filum. Clip placement was optimized to allow watertight dural closure. Final micro-Doppler examination and ICG angiography confirmed occlusion of the fistula. The dura was closed with running 6-0 Prolene in standard fashion.

### 5:51 Disease Background and Takeaways.

This rare type of vascular lesion can be challenging to treat if it is not recognized prior to treatment. Despite the classification schema available, both endovascular and surgical approaches must be weighed with patient clinical and anatomical factors in mind.

The size of the venous pouch, its proximity to the conus, and the associated mass effect in the spinal canal on the surrounding nerve rootlets made surgical manipulation for adequate dissection and visualization challenging. To achieve cure, the fistula must be completely disconnected from the venous side. Based on the MRI and angiographic imaging, it was suspected that the fistula would be related to the conus medullaris, and the filum terminale relationship was not identified until numerous primary arterial feeders were ligated and adequate dissection for visualization was achieved. Once the nature of the fistula at the level of the filum terminale was demonstrated, the surgical goals were refined and the fistula was quickly occluded. With this in mind, despite filum terminale arteriovenous fistulas accounting for < 5% of all spinal fistulas, it is important to consider all potential pathological variants in this anatomical territory, and, in retrospect, based on the imaging findings suggesting the fistula occurring at L2 (below the level of conus), the nature of the fistula could have been established prior to surgical treatment.^3–5^

### 7:07 Disease Background and Takeaways Continued.

Beside performing a postoperative spinal angiogram, an additional option would be to perform it intraoperatively. But we believe to have had adequate visualization of the anatomy with the ICG videoangiogram. Although performing an intraoperative spinal angiogram in the prone position is challenging, it would have been an option if ICG capability was unavailable.

It would be preferable to coagulate and divide the vein following clip application, to avoid leaving permanent clips in the dura. The dura was challenging to close, and clips in that region could cause undue mass effect. However, several factors were considered for the decision to leave permanent clips. The mass of the venous varix made visualization of the filum challenging, and the vein appeared thin walled and friable. To coagulate and divide the vein without identification of the filum at all times was potentially unsafe given the proximity to the conus and motor rootlets. To avoid mass effect on the conus, the body of the clips were placed caudally and they were angled appropriately to primarily close the dura.

### 8:04

Postoperative spinal angiogram was performed demonstrating complete occlusion of the fistula with venous filling only seen in the epidural venous plexus.

Postoperatively, the patient remained on flat bedrest for 24 hours to optimize dural healing and avoid postural headache. The patient demonstrated improvement in right lower extremity strength and parasthesias immediately after surgery, and demonstrated further improvement on short-term and 3-month outpatient follow-up with resolution of symptoms and improved gait. One-year follow-up diagnostic spinal angiogram is planned, followed by periodic MRI for surveillance.

Thank you.

## Disclosures

The authors report no conflict of interest concerning the materials or methods used in this study or the findings specified in this publication.

## Author Contributions

Primary surgeon: Gould. Assistant surgeon: Koutsouras. Editing and drafting the video and abstract: Koutsouras, Bashir. Critically revising the work: all authors. Reviewed submitted version of the work: all authors. Approved the final version of the work on behalf of all authors: Koutsouras. Supervision: Gould.

## Supplemental Information

### Patient Informed Consent

The necessary patient informed consent was obtained in this study.
